# Physical Health and Health Behaviours of Australians with Psychosis

**DOI:** 10.1007/s10597-024-01417-w

**Published:** 2025-02-20

**Authors:** Brenda Happell, Chris Platania-Phung, Trentham Furness, Brett Scholz, Theo Niyonsenga, Andrew Watkins, Jackie Curtis, Zijian Wang, Supriya Khanijou, Robert Stanton

**Affiliations:** 1 Faculty of Health,, Southern Cross University,, Lismore, Australia; 2 School of Nursing and Midwifery, The University of Newcastle, Callaghan, Australia; 3 Equally Well, Australia, Orange, Australia; 4 Forensicare, Melbourne, Australia; 5 School of Medicine and Psychology, Australian National University, Canberra, Australia; 6 Health Research Institute, University of Canberra, Canberra, Australia; 7 Mindgardens Neuroscience Network, Sydney, Australia; 8 Discipline of Psychiatry and Mental Health, University of New South Wales, Sydney, Australia; 9 Deakin University, Geelong, Australia; 10 Cluster for Resilience and Wellbeing, Appleton Institute, School of Health, Medical and Applied Sciences CQUniversity, Rockhampton, Australia

**Keywords:** Nurse-led, Physical health, Cardiovascular diseases, Metabolic syndrome, Mortality, Equally well

## Abstract

People living with psychosis live up to 20 years less compared to the general population. Cardiometabolic ill-health and barriers to health-related behaviour are significant contributors. This is a cross-sectional descriptive study of cardiometabolic health and health behaviours of consumers attending a public community mental health service in an Australian city. One hundred and fourteen consumers currently living with psychosis participated. Standard measures of cardiometabolic health, quality of life and, health-related behaviours were utilised. Data were analysed using descriptive statistics. The cohort reported higher fruit intake and physical activity, and lower excess alcohol use compared to previous studies. Health-related behaviours including smoking and vegetable intake were poorer than previously reported. Participants had low levels of cardiometabolic health (e.g. abnormal lipids). Physical and mental quality of life was also lower than for general populations. Improved efforts to address physical health for people with mental health conditions are urgently needed.

## Introduction

Psychosis is a mental illness that may be characterised by significant disability (GBD 2019 Mental Disorders Collaborators, 2022). A recent systematic review identified that people living with enduring psychosis (heroin people living with psychosis) may experience reduced global functioning, social isolation, low employment, and quality of life, resulting in a substantial disease burden (Korman et al., 2023). The median lifetime prevalence of adults living with psychosis is estimated 7.4 per 1000 but varies widely based on study setting and methodological quality (Moreno-Küstner et al., 2018). People living with psychosis experience early mortality of 10 to 20 years, compared to the general population (Peritogiannis et al., 2022; Plana-Ripoll et al., 2019). Known contributors to this significant mortality gap include poor physical health behaviors such as low levels of physical activity, nutritional inadequacies, and smoking (Firth et al., 2019; Hahn et al., 2018). Other systemic contributors include poor access to physical health care (Lawrence & Kisely, 2010), inequity in financing for mental compared to physical health care (O’Connell et al., 2022), medication side effects (Pillinger et al., 2020; Schneider-Thoma et al., 2019), and socioeconomic status (Liu et al., 2017).

Improving poor physical health of consumers is also supported among national (National Mental Health Commission, 2018) and international (World Health Organization, 2021) strategies and guidelines. Most importantly, improving physical health has been identified as a priority for consumers (Aboaja et al., 2021). Mental health nurses have been actively involved in research and practice initiatives to improve physical care and treatment and are well positioned to work closely with consumers in addressing their physical health needs (Happell et al., 2023) and support a consumer’s recovery journey (McKenna et al., 2014). Consumers have expressed support for a mental health nursing role dedicated to physical health (Happell et al., 2016) and have positively evaluated this role in a recent qualitative research project (Tabvuma et al., 2023).

## Background

Metabolic syndrome, a cluster of inter-related risk factors for cardiometabolic diseases including type 2 diabetes and heart disease (Alberti et al., 2006), is common for people living with psychosis (Bioque et al., 2018; Cocoman & Gallagher, 2019) independent of antipsychotic medication use (Garrido-Torres et al., 2021). In addition to its contribution to early mortality, metabolic syndrome negatively impacts quality of life of people living with psychosis (Foldemo et al., 2014; Korman et al., 2023).F.

Increasingly, lifestyle interventions which adopt a whole-of-person perspective of primary prevention and treatment are being implemented as non-pharmacological strategies to address the poor physical health of people with mental health concerns including those living with psychosis (Firth et al., 2020). Such approaches include physical activity, dietary, sleep, and smoking cessation interventions which may be individually tailored and delivered to consumers according to their needs. In particular, such interventions delivered by specialist nursing roles have been shown to improve health outcomes for people with mental health concerns including psychosis (Furness et al., 2020; Happell et al., 2012, 2018; Happell & Millar, 2014; McKenna et al., 2014). Nurses are well placed to develop and deliver such interventions as a result of their strong therapeutic relationship with consumers and their educational preparation for physical health care (Happell et al., 2011; Tabvuma et al., 2022).

However, despite policy and research efforts to improve the physical health and physical health care of people diagnosed with mental illness including those living with psychosis, the mortality gap remains, and broad implementation of integrated physical health and mental health care has remained elusive. Previous Australian studies showed a high prevalence of risk factors for cardiometabolic disease in people living with psychosis including hypertension, obesity, and abnormal lipids profiles. (Galletly et al., 2012; Morgan et al., 2013). These risk factors were accompanied by unhealthy behaviors such as low levels of physical activity, poor fruit and vegetable intake, and high prevalence of smoking and substance use (Galletly et al., 2012; Jankowski et al., 2022; Morgan et al., 2013). Some of these behaviours were associated with metabolic syndrome. Moreover, recent studies in Western Australia suggested little change over time in physical health care provision and indeed a worsening in the prevalence of metabolic syndrome (Morgan et al., 2021).

Mental health nurses, while well suited to addressing physical health concerns, experience barriers in providing this care, including lack of knowledge, skills and confidence (Happell et al., 2013; Voort et al., 2024) and the services focus on mental illness as core business (Happell et al., 2023) which frequently results in physical health needs not being adequately addressed. Acknowledging these limitations led to the development and implementation of a specialist nursing role specifically addressing physical health (Happell et al., 2019).

Despite existing knowledge on the poor physical health and health behaviours among people with mental health conditions, it remains important to continue to examine this phenomenon to reinforce to health care systems and policy makers of the need to improve physical health care. Therefore, this study examines physical health and health behaviours among consumers living with psychosis attending a large community mental health service in an Australian capital city. We include a novel measure of mean arterial pressure (MAP), since this marker is known to be associated with cardiovascular and cerebrovascular diseases along with standard health and behaviours measures (Gao et al., 2023).

This research is of particular importance to the health service, who have identified physical healthcare as a treatment priority and seek to establish a baseline for physical health parameters in this at-risk population. No previous such studies have been conducted hence the findings may inform health service redesign and intervention implementation. Moreover, the findings may strengthen the case for physical health intervention as part of usual care, and reinforce policy directives to improve physical health care to reduce the early mortality and disease burden among this population.

## Methods

### Research Design

This cross-sectional descriptive study presents cardiometabolic health and health behaviour data on consumers living with psychosis attending a large community mental health service in an Australian capital city. The study received ethics approval from the ACT Health Research and Ethics Committee (ETH.3.18.057), and all participants provided written informed consent.

### Study Setting

### Participants

All English-speaking consumers aged 18–65, diagnosed with a DSM-5 psychotic disorder attending the service were approached to participate by study staff. Following written consent, participants underwent screening based on the measures described below.

### Outcome Measures

Sample demographic characteristics (e.g., age and gender) were collected along with cardiometabolic health and health behaviours assessments using standard blood markers, anthropometric characteristics, blood pressure, smoking status and frequency, dietary and physical activity status, number of comorbid conditions, and quality of life. Blood markers (i.e., total cholesterol, LDL, HDL, triglycerides, lipid profile and glycosylated haemaglobin (HbA1c), were assessed from fasting venous samples drawn from the antecubital vein by an experienced nurse and analysed by a commercial pathology laboratory. Anthropometric characteristics (i.e., body mass index (BMI) and waist circumference measured perpendicular to the long axis of the trunk at the narrowest point between the lower costal border and the iliac crest ) were determined using standard procedures (Norton & Olds, 1996). Blood pressure was taken on the dominant upper arm using a digital sphygmomanometer and reported using the standard convention (systolic / diastolic). We then classified people with hypertension as those whose systolic blood pressure was greater ≥ 140 mmHg, or a diastolic blood pressure ≥ 90 mmHg (National Heart Foundation of Australia, 2016). To further understand haemodynamic function, we calculated mean arterial pressure, a major determinant of tissue perfusion (Papaioannou et al., 2016), using the following equation: MAP= [(2 x diastolic) + systolic] / 3 (Smeltzer et al., 2010).

Health behaviors were assessed in accordance with the National Health Survey (Australian Bureau of Statistics, 2015). Consumers self-reported their smoking status (Yes/No) and smoking frequency (At least once daily [Yes/No], At least once weekly [Yes/No]). Dietary status was examined using questions related to daily consumption of fruits and vegetables (number of serves), discretionary food (e.g., take away), and consumption of sugary drinks. Alcohol use was examined according to frequency of consumption (Never – 4 + times per week), number of standard drinks consumed per session (1–2–10+) and frequency of binge drinking, defined as consuming≥5 drinks/session (Never – Daily or almost daily). Physical activity participation was examined using the Active Australia Questionnaire (AIHW, 2003). Participants were classified as sedentary (0 min of activity per week) insufficiently active (1–149 min of activity per week) or sufficiently active (≥ 150 min of activity per week), based on total minutes of activity (walk time + moderate activity time + (2 x vigorous activity time), in accordance with guidelines (AIHW, 2003). Participants’ self-reported physical health comorbidities are reported using proportions and number of comorbid conditions. Quality of life was measured using the 35-item Assessment of Quality of Life (AQoL)-8D instrument (Richardson et al., 2011). The AQoL-8D is shown to be sensitive for use in mental health populations (Richardson, Elsworth, Richardson et al., 2011a, b) with high internal reliability (α = 0.96) and good content validity (Richardson et al., 2014). The instrument is a multi-attribute utility instrument assessing eight dimensions of mental and physical health, which can be collapsed into two super dimensions. Five dimensions (mental health, happiness, coping, relationships, and self-worth) form the mental super-dimension while three dimensions (independent living, pain, senses) form the physical super-dimension. In the present study, unweighted mean scores for each dimension are presented since the scoring is intended to present the psychometric score for health-related quality of life, as opposed to using weighted scores which are used to derive utility scores for economic analyses.

### Statistical Analysis

Data are reported using descriptive statistics (mean, standard deviation, frequency, proportion) as appropriate. For between group comparisons, independent samples *t*-tests were conducted. For AQoL-8D dimensions, mean scores were compared to population norms using 1-sample *t*-tests. All *p*-values are 2-sided. Data analysis was conducted using Statistical Package for the Social Sciences (SPSS V28, IBM Corp, Armonk NY).

## Results

In total, 114 participants (64 males) volunteered for this study and provided written informed consent. Mean age of participants was 42.3±10.7 years. Females were on average, significantly older than males included in this study (t(109) = 1.991, *p* < 0.05). Demographic characteristics of participants are presented in Table 1. Over half (*n* = 51, 53.7%) had attended secondary school, while less than one-third (*n* = 28, 28.9%) were employed. A substantial proportion (*n* = 69, 71.9%) were receiving a disability support pension.

**Table 1:** Participant demographic characteristics (*n* = 114)

Characteristic		
Age	Mean	SD
	42.3	10.7
Gender*	n	Percent
Male	64	56.1
Female	47	41.2
Missing	3	2.6
Education^#^		
Primary school	1	0.9
Secondary school	51	53.7
Vocational education	21	22.1
Tertiary education	22	23.2
Missing	19	16.7
Employment status		
Currently employed	28	24.6
Disability support pension (*n* = 96)	69	71.9
Missing	17	14.9

*Three participants did not provide a response to the question about gender

^#^Refers to highest level of education attained

### Cardiometabolic Health

Cardiometabolic and disease comorbidity data are shown in Table 2. Lipid profile data were not available for all participants, however, from those for whom data were available, most had abnormal lipid parameters. Almost all (*n* = 98, 92.5%) had HbA1c results below the general target of ≤ 7%. Most participants (81.2%) were classified as overweight or obese (BMI > 25 kg/m²) and almost all females and three-quarters of males had waist circumference measurements placing them at elevated risk of cardiovascular disease (≥ 80 cm for females; ≥94 cm for males). There were significant between-gender difference in BMI, with BMI being significantly higher for females compared to males (t(104) = 2.208, *p* < 0.05, mean Dif = 3.1 kg.m^− 2^).

**Table 2:** Cardiometabolic health of Australian adults with psychosis

Metabolic profile (total sample)	Mean	SD
Cholesterol (*n* = 15)	4.9	0.9
HDL (*n* = 95)	1.3	0.6
LDL (*n* = 92)	2.6	0.9
Triglycerides (*n* = 11)	1.8	0.9
Abnormal lipids	n	Percent
High cholesterol (> 4.0) (*n* = 15)	14	93.3
Low HDL (< 1.0) (*n* = 95)	30	31.6
High LDL (> 1.8) (*n* = 92)	77	83.7
High triglycerides (> 2.0) (*n* = 11)	3	27.3
HbA1c (*n* = 106)	Mean	SD
	6.0	2.6
Body Mass Index (*n* = 106)	Mean	SD
Group	31.9	7.3
Males (*n* = 61)	30.6	6.5
Females (*n* = 45)	33.7	8.1
Waist Circumference (*n* = 103)	Mean	SD
Group	109.8	19.5
Males (*n* = 60)	108.3	19.1
Females (*n* = 43)	112.0	20.1
Haemodynamic status (*n* = 111)	Mean	SD
Hypertensive (*n* = 111)	29	26.1
Mean Arterial Pressure (*N* = 111)	mean	SD
	95.6	11.8
Within healthy range	n	Percent
	98	88.3
Comorbidity	n	Percent
Has a comorbid condition (Yes)^^^	79	69.3
Heart disease	1	0.9
Osteoarthritis	9	7.9
Hypertension	18	15.8
Depression	64	56.1
Diabetes (Type I or II)	12	10.5
Other conditions^*^	41	36.0
Number of comorbid conditions	Mean	SD
	1.3	1.3

^^^ Numbers add to more than 79 due to multimorbidity

^*^ Other conditions (free text response) included hepatitis c, anaemia, thyroid disease, respiratory conditions, unspecified orthopaedic conditions, epilepsy, and vascular conditions

From 111 participants from whom blood pressure readings were obtained, 29 (26.1%) were classified as hypertensive based on a systolic/diastolic blood pressure of > 140/90mmHg. Average MAP was 95.5 ± 11.8mmHg and *n* = 98 (88.3%) had a MAP within the normal range. Finally, we recorded physical health comorbidity. Over two-thirds (*n* = 79, 69.3%) reported at least one comorbid physical health condition with the mean number of conditions reported as 1.3 ± 1.3 (range 0–7).

### Health Behaviors

Outcomes for health behaviors are shown in Table 3. Almost half of all participants (49.1%) responded that they smoked tobacco, with *n* = 53 (96.4%) stating they smoke at least once daily, and a further two indicating they smoke at least once weekly. More than one-third (*n* = 43, 38.4%) indicated they met the dietary guidelines of two or more serves of fruit per day. Almost one-third (*n* = 33, 29.8%) reported eating one serve of fruit per day, while *n* = 9 (8.0%) reported not eating fruit at all. Only four participants (3.6%) indicated they met the dietary guidelines for vegetable intake (min 5 serves/day). Almost one-quarter (*n* = 25, 22.3%) stated they consumed on average less than one serve of vegetables per day while seven (6.3%) stated they did not eat vegetables at all. Over half (*n* = 57, 50.9%) reported abstinence from alcohol. Most commonly, respondents reported drinking alcohol monthly or less (*n* = 29, 25.9%, while a small proportion (*n* = 8, 7.1%) reported alcohol use on 4 or more occasions per week. Drinkers most commonly reported consuming 1–2 drinks per occasion, with a small number (*n* = 3, 5.5%) reporting consuming 10 or more drinks per occasion. Most drinkers (*n* = 22, 40.7%) reported never consuming ≥ 5 drinks on any one occasion, however, *n* = 2 (3.7%) reported hazardous drinking daily or almost daily. Almost two-thirds (*n* = 73, 64%) reported meeting the public health guidelines of 150 min of physical activity per week, while only a small number (*n* = 6, 5.3%) reported being sedentary (0 min of physical activity per week).

**Table 3:** Physical health behaviours

Smoking (*n* = 112)	*n*	Percent
Yes	55	49.1
No	57	50.9
Smoke regularly (at least once per day)	53	96.4
Smoke regularly (at least once per week)	2	3.6
Fruit intake (*n* = 112)	n	Percent
Do not eat fruit	9	8
Less than one serve	31	27.7
1 serve	29	25.9
2 serves	22	19.6
3 serves	14	12.5
4 serves	6	5.4
5 serves	1	0.9
Vegetable intake (*n* = 112)	n	Percent
Do not eat vegetables	7	6.3
Less than one serve	25	22.3
1 serve	33	29.5
2 serves	19	17.0
3 serves	16	14.3
4 serves	8	7.1
5 serves	2	1.8
6 serves or more	2	1.8
Alcohol intake (*n* = 112)	n	Percent
Frequency		
Never	57	50.9
Monthly or less	29	25.9
2–4 times per month	8	7.1
2–3 times per week	10	8.9
4 + times per week	8	7.1
Number of standard drinks per session		
1–2	21	38.2
3–4	19	34.5
5–6	8	14.5
7–9	4	7.3
10 +	3	5.5
Frequency of binge drinking (≥ 5 drinks per session)		
Never	22	40.7
Less than monthly	17	31.5
Monthly	9	16.7
Weekly	4	7.4
Daily or almost daily	2	3.7
Physical activity (*n* = 114)	n	Percent
Sedentary	6	5.3
Insufficiently active	35	30.7
Sufficiently active	73	64

### Quality of Life

Quality of life scores for each dimension including super dimension scores, and comparison with population norms are shown in Table 4. Notably, all mean scores were significantly lower than population norms.

**Table 4:** AQoL-8D score (*n* = 112)

AQoL-8D dimension	Minimum	Maximum	Mean ^*^	SD	Population norms ^1^
Independent Living	4	17	8.23	3.26	92.50
Happiness	3	20	11.03	3.27	71.38
Mental Health	9	37	21.04	5.64	76.09
Coping	4	15	8.47	2.15	74.56
Relationships	7	32	16.31	4.74	83.76
Self Worth	3	15	7.97	2.74	79.16
Pain	1	14	6.29	2.97	85.40
Senses	2	11	5.89	1.80	88.29
Physical Super dimension	10	38	20.41	6.50	89.43
Mental Super dimension	35	103	64.82	15.98	77.59

^*^ All mean scores significantly lower than population norms

^1^ Population norms derived from Maxwell, A., Özmen, M., Iezzi, A., & Richardson, J. (2016). Deriving population norms for the AQoL-6D and AQoL-8D multi-attribute utility instruments from web-based data. Quality of Life Research, 25(12), 3209–3219. 10.1007/s11136-016-1337-z

## Discussion

The present paper describes cardiometabolic health and health behaviour characteristics of a cohort of Australian adults living with psychosis who received care in a public community health service. Cardiometabolic descriptors mostly fall into high risk categories and are consistent with collated findings among cohorts with a diagnosis of psychosis (Ma et al., 2021). With the exception of physical activity, health behaviour characteristics were mostly poor, suggesting poor self-care in the physical health domain, also consistent with the literature (Atakan et al., 2023; Kim et al., 2019; Martland et al., 2023b). However, it is important to acknowledge that other factors may impact health behaviours including engagement with health services (Kim et al., 2019), therapeutic relationships (Tabvuma et al., 2022), health service priorities (Happell et al., 2012), or socioeconomic status (Liu et al., 2017).

Interestingly, BMI was significantly higher for females compared with males. This finding aligns with the Australian data indicating females with mental health issues present with greater prevalence of physical health comorbidity compared with males (Australian Institute of Health and Welfare, 2024). Similarly, antipsychotic medication treatment frequently results in weight gain and is common among people living with psychosis (Wu et al., 2022) and emerging data suggests that there are sex differences in gene expression associated with this type of weight gain (Sainz et al., 2019).

Harmful tobacco smoking is common among people living with psychosis with rates between 17% (Cooper et al., 2012; Li et al., 2017), and 34% (McKenna et al., 2014). Previous data suggest around three-quarters of people living with psychosis report being a current smoker, and a lifetime prevalence of 81% (Cooper et al., 2012). Given smoking is a contributor to premature mortality in people living with psychosis, identifying access to feasible, acceptable, and effective interventions targeting smoking reduction or cessation should be prioritized. Evidence suggests both pharmacological (Siskind et al., 2020) and m-health (Sawyer et al., 2022) approaches may be suitable, but that interventions should be tailored specifically to the needs of people living with psychosis. In the present study 49% of consumers reported smoking. This is significantly greater than the 11% reported for the Australian general population (Wakefield et al., 2023), but is approximately 50% lower than that reported in recent international studies on people living with psychosis (Rogers et al., 2024).

The current study also identified poor nutritional behaviors regarding fruit and vegetable intake, aligning with existing literature (Aucoin et al., 2018; González-Rodríguez et al., 2023). This may be attributed to ongoing need for health literacy education (Haidl et al., 2019) and may also be influenced by contextual factors such as socio-economic status, cost associated with healthy eating (Martland et al., 2023) and food security/availability (Giles et al., 2023; Teasdale et al., 2023). Notably, low alcohol consumption was self-reported in the current study, which is inconsistent with the extant literature (Archibald et al., 2019). Psychotic disorders are often associated with alcohol use disorders (Archibald et al., 2019), with the combined impact, extremely deleterious to overall health (Seeman, 2023). The current findings regarding alcohol intake, while welcome, may be underpinned by social desirability bias (Jankowski et al., 2022), or could be a consequence of the recent COVID-19 pandemic, which saw Australians consume less alcohol due to the closure of licensed premises and the implementation of social distancing (Callinan et al., 2021; Greenwood et al., 2023).

Previous reviews and studies highlight low physical activity in consumers living with psychosis (Bradley et al., 2022; Stubbs et al., 2016; Tew et al., 2023), despite the reported importance of physical activity among this cohort (Swora et al., 2022; Ziebart et al., 2022). In contrast, the present study reports around two-thirds of participants met the Australian Physical Activity Guidelines. This may be explained, at least partly by the higher levels of physical activity in the region in which the study was undertaken, or the well-established concern of social desirability bias when using self-report measures of physical activity (Australian Bureau of Statistics, 2018).

### Quality of Life

Recent studies have demonstrated the utility of the AQoL-8D in people with mental health disorders including responsiveness to change over time, and the absence of floor or ceiling effects (Chan et al., 2022). While dimension and super-dimension norms are not available for the population included in the present study, our findings demonstrate mean scores to be significantly lower across all dimensions compared to population norms (see Table 4) The reasons for this disparity could be related to the impact of the recent COVID-19 pandemic. Other studies have reported poor QoL in people living with psychosis, which may be a consequence of low functioning, loneliness, or medication side effects (Germain et al., 2019).

The benefits of community mental health services in the provision of healthcare for consumers has been demonstrated through interventions describing healthcare literacy (Munawar et al., 2022), blood pressure and vaccination status (Bartels et al., 2014), and nutritional intake and physical activity (Bressington et al., 2018). Success of future community based interventions should consider barriers to improving physical health and health behaviours such as mental illness stigma (De Hert et al., 2011), consumer disengagement (Powell et al., 2021), reluctance (Bartlem et al., 2018), and amotivation (Every-Palmer et al., 2018). Nurses are potentially well placed to support and/or lead such interventions due to their highly developed interpersonal communication skills and knowledge of physical healthcare and health behaviours (Happell et al., 2023; Robson & Haddad, 2012).

### Nurse-Led Physical Health Interventions

Findings of the current study suggest nurse-led physical health interventions and clinical practices should be supported among community mental health services to identify consumers at risk of cardiometabolic and cardiovascular diseases. This may involve development of physical health policy and guidelines (National Mental Health Commission, 2018), allocation of resources (Suggett et al., 2021), and nurse-led physical health interventions to improve consumer physical health (Happell et al., 2024).

Nurse-led interventions to support positive lifestyle behaviour changes have been shown to be effective for people that experience psychosis (Happell et al., 2011, 2023). Nurses in mental health settings have led individual and group smoking reduction intervention involving counselling, medication management (Happell et al., 2023), and facilitated reduced smoking rates when support is integrated to lifestyle/ healthy behaviour programs (Curtis et al., 2018; Stanley et al., 2020). Therefore, nurses have a significant role to play in smoking reduction programs for people with mental health conditions including psychosis. Incorporated into holistic healthy lifestyle interventions, nurses in partnership with consumers have led programs to improve nutritional behaviours (Fernandez Guijarro et al., 2019), reduce harmful drug and alcohol consumption (Harney et al., 2021; Meepring et al., 2018), facilitate increased physical activity (Celik Ince & Partlak Gunusen, 2021; Fraser et al., 2018), and moderate other metabolic risk factors (Happell et al., 2023). Introducing a specialist physical health nursing role to coordinate this physical health care has been shown to be successful and should be considered as a strategy going forward (Tabvuma et al., 2023).

### Limitations

The findings presented are not without limitation. Of the approximately 400 eligible consumers, a sample of 114 represents approximately 28%. We do not have data on those who chose not to participate and therefore can only speculate on the reasons for non-participation. However, these could include that they received physical health monitoring elsewhere (for example their General Practitioner), social desirability bias, a low level of interest in physical health assessments, or concerns regarding how adverse findings such poor physical health outcomes may have impacted their care.

Blood markers were not available from all participants meaning interpretation of cardiometabolic status should be done with caution. It is not clear why reporting of cholesterol or triglycerides are absent in comparison to HDL and LDL data, however this could be due to analyses being requested or undertaken at the time, the existence of recent routine test data not aligning with the present study, not justifying further analysis or consumer refusal for some tests to be undertaken. This is markedly different from large scale population studies such as the Rotterdam study where complete datasets were available from the complete cohort. In contrast, we note other studies report results of blood marker which represent only a small proportion of the study sample (Alonso et al., 2022; Çakici et al., 2023). Routine blood monitoring is critical to manage cardiometabolic health and medication status in consumers living with psychosis and future studies should ensure comprehensive collection of such data. The present study did not collect medication status and some who may have returned normal range blood marker or haemodynamic outcomes may have been masked by pharmacological interventions, for example for diabetes or hypertension. Taken together this meant the presence of metabolic syndrome could not be definitively determined. Some data may be impacted by social desirability bias or recall bias. For example, the high proportion of participants meeting physical activity guidelines is inconsistent with other studies and could be a consequence of seeking approval from the data collection team. Alternatively, it could be a consequence of selection bias in that people willing to participate in a physical health intervention may already have some healthy behaviors.

## Conclusion

The present study shows that despite policy recommendations to deliver interventions to reduce early mortality and improve physical health in people with mental health conditions including psychosis, poor physical health remains highly prevalent. While it can be acknowledged that policy implementation is a wicked problem, requiring the coordination of systemic and individual factors and multiple decision makers, much more needs to be done to achieve parity in physical health care. Nurses have demonstrated their capacity to lead such interventions due to their therapeutic relationship with consumers. Moreover, nurses should commence or continue their active engagement in physical health interventions as an opportunity to further strengthen their therapeutic relationship with consumers. Interventions such as walking programs or regular physical health check do not need to be resource intensive and should form part of routine care to reduce to poor physical health experienced by this cohort of mental health consumers.

### Relevance for Clinical Practice

These findings highlight that people living with psychosis who access community mental health services continue to exhibit poor cardiometabolic health. National-level equity-based policies that centre on the need for effectively combining mental and physical healthcare services require urgent implementation to address this disparity (Australian Bureau of Statistics, 2018; National Mental Health Commission, 2018; Te Pou, 2022). Evidence-based, nurse-led, integrated programs coupled with co-designed care approaches that suit the site-specific intricacies of day-to-day practice may help bridge this gap (Stanley et al., 2020). Such role-based integrated care may be a promising avenue for change since principles can apply across contexts for comprehensive care which leverages good therapeutic relationships and continuity of care. Furthermore, integrated care roles may better ensure that the range of risks, lifestyle factors evidenced in the current study (e.g. abnormal lipids, smoking) are *concurrently* attended to in conjunction with quality-of-life considerations, in partnership with consumers. Mental health services should prioritise the development and delivery of co-designed physical and mental health services to meet the complex needs of people living with psychosis.
